# In Vivo Evaluation of Efficacy and Toxicity of Bioinspired Peptides With Antimicrobial Properties Against Fungal Infection in *Galleria mellonella* Larvae

**DOI:** 10.1155/ijm/7272293

**Published:** 2026-07-11

**Authors:** Allan da Silva Conceição, Lorena Mendes dos Santos, Gabriel Bonan Taveira, Valdirene Moreira Gomes, André de Oliveira Carvalho

**Affiliations:** ^1^ Laboratório de Fisiologia e Bioquímica de Microrganismos, Centro de Biociências e Biotecnologia, Universidade Estadual do Norte Fluminense Darcy Ribeiro, Campos dos Goytacazes, Rio de Janeiro, Brazil, uenf.br

**Keywords:** antifungal, antimicrobial peptides, opportunistic fungus, toxicity

## Abstract

**Background:**

Fungal infections, particularly those caused by *Candida* species, represent a growing public health threat due to increasing resistance to conventional antifungal agents. Consequently, there is a need for the development of novel antifungals.

**Objective:**

This study evaluated the in vivo toxicity and efficacy of three peptides with antimicrobial properties, RR, D‐RR, and WR that exhibit in vitro antifungal activity and are bioinspired from the *Vigna unguiculata* defensin *γ*‐core.

**Methods:**

Using *Galleria mellonella* larvae, infections were established with a clinical strain of *Candida tropicalis* and *Candida albicans* over a 144‐h period. Toxicity and efficacy were evaluated through survival assays, hemocyte density measurements, fungal load quantification, and health index monitoring. Peptides were administered in multiple doses over time.

**Results:**

Fungal lethal doses (FLD) in *G. mellonella* were 1 × 10^6^ cells/mL for *C. tropicalis* and 0.5 × 10^6^ cells/mL for *C. albicans*. Peptide lethal doses were 41 *μ*M (RR) and 32 *μ*M (D‐RR) for *C. tropicalis*, and 60 *μ*M (WR) for *C. albicans*. All treatments were nontoxic. Survival of infected larvae was 93.3% with RR and D‐RR. Among peptides, WR showed the greatest efficacy, achieving 100% survival and outperforming fluconazole, while supporting continued larval development. The peptides exerted inhibitory effects within the in vivo tissue environment of *G. mellonella*.

**Conclusions:**

These findings indicate that the peptides are promising safe antifungal candidates, warranting further investigation into their mechanisms of action and stability.

## 1. Introduction

Invasive mycoses have seen a substantial rise in recent years, with serious fungal infections affecting an estimated 6.5 million people worldwide and causing approximately 3.8 million deaths annually [1–4]. This high incidence of invasive mycoses and associated mortality is linked to multiple factors. One of the primary risk factors is the increase in immunocompromised individuals, a result of medical advances, new surgical techniques, and medications that enhance patient survival. This group includes people living with acquired immunodeficiency syndrome [5], patients undergoing cancer and corticosteroid treatments, transplant recipients on immunosuppressants, and the elderly [6]. Climate change is another significant factor. Emerging evidence indicates that rising global temperatures are driving fungal adaptations to warmer conditions [7] leading to the emergence of new fungal pathogens such as *Candida auris* [8] and also causing an expanded geographic distribution of fungal infections [9]. These trends are compounded by additional challenges. One major obstacle is drug development, the evolutionary similarity between fungi and animals, which makes it difficult to identify unique molecular targets for antifungal drugs [10]. This similarity partly explains the limited number of clinically available antifungals [11]. Compounding this problem is the growing resistance of fungi to existing antifungal drugs, further restricting treatment options [12]. Unlike many pathogens, fungi can survive independently in the environment, meaning they are not subject to the virulence attenuation that occurs in pathogens reliant on hosts for survival [8].

Given the urgent need for novel antifungal agents, antimicrobial peptides (AMPs) have emerged as promising candidates. We define a peptide as antimicrobial if it has been experimentally validated to inhibit microbial growth [13]. These evolutionary conserved molecules are produced by a wide range of organisms, in bacteria, to reduce competition, and in metazoans as part of their innate immunity [14]. Typically composed of up to 100 L‐amino acid residues, AMPs are ribosomally synthesized, amphipathic, and carry a net positive charge at physiological pH. Their broad‐spectrum antimicrobial activity makes them attractive for therapeutic development [15]. In this study, we investigated three bioinspired peptides with antimicrobial properties, RR, D‐RR and WR, strategically modified to enhance charge and hydrophobicity, based on the *γ*‐core motif of *Vigna unguiculata* defensin. These designed features are aimed at improving microbial inhibition while minimizing cytotoxicity to mammalian cells [16]. The peptides exhibited potent antifungal activity against opportunistic dimorphic fungi, with low toxicity profiles [16]. We further explored their mechanism of action against two clinical isolates of *Candida tropicalis* and *Candida albicans* [17, 18]. The hypothesis of this study was that the three peptides, which had previously demonstrated antifungal activity in vitro, would also exhibit antifungal activity and show no toxicity in vivo by controlling fungal infection in *Galleria mellonella*.

## 2. Materials and Methods

### 2.1. Synthetic Peptides

The three peptides were derived from a previously designed peptide, A_36,42,44_ *γ*_32–46_VuDef, abbreviated as DD, which is based on the *γ*‐core region of Vu‐Def1. DD consists of a 15–amino acid sequence (L_32_SGRARD_38_D_39_VRAWATR_46_, numbered based on Souza et al. [19]). The designation *γ*_32–46_ refers to the *γ*‐core region of Vu‐Def1. In this design, cysteine residues in the original sequence were substituted with alanine at positions 36, 42, and 44 to prevent the formation of disulfide bridges, which are absent in the native molecule; this modification is indicated as A_36,42,44_ in the peptide name. The abbreviation “DD” reflects the presence of two consecutive aspartic acid residues at positions 37 and 38. DD has a net charge of +2 and a hydrophobicity value of +21.48 kcal/mol (with higher values indicating greater hydrophilicity according to the Wimley–White scale). This peptide retains antimicrobial activity against *Leishmania amazonensis*, comparable to that of the full‐length Vu‐Def1 defensin, and exhibits a partially characterized mechanism of action involving destabilization of the protozoan plasma membrane [16, 19]. Building on this design, new peptides were developed to optimize biochemical properties such as net charge, hydrophobicity, and chirality. These parameters are critical for the interaction of peptides with antimicrobial properties with fungal cell membranes. Based on this approach, three derivatives were generated: RR (A_36,42,44_R_37,38_ *γ*_32–46_VuDef): Aspartic acid residues at positions 37 and 38 (negatively charged) were replaced with arginine residues (positively charged), increasing the net charge from +2 to +6. This substitution also reduced hydrophobicity, yielding +18.32 kcal/mol. D‐RR (D‐A_36,42,44_R_37,38_ *γ*_32–46_VuDef): The enantiomeric form of RR, composed of amino acids in the D configuration. This modification confers increased resistance to proteolytic degradation and reduced cytotoxicity in human cells, while maintaining antimicrobial activity and preserving the charge and hydrophobicity profile of RR. WR (A_42,44_R_37,38_W_36–39_ *γ*_32–46_VuDef): designed to increase hydrophobicity through the introduction of tryptophan residues at positions 36 and 40, resulting in reduced hydrophobicity of +14.10 kcal/mol. This property favors stronger interactions with fungal membranes (Figure S1).

RR, D‐RR, and WR were synthesized, dissolved in sterile ultrapure water at 2 *μ*g/*μ*L (approximately 1000 *μ*M, depending on the peptide molecular weight), and stored as aliquots at −70°C [16].

### 2.2. Fungi

The fungal strains *C*. *tropicalis* ATCC 750 and *C*. *albicans* SC 5314/ATCC MYA‐2876, here referred to as *C. albicans* SC 5314 for simplicity, were cultivated in Sabouraud′s medium (10‐g/L meat peptone, 5‐g/L casein peptone, 20‐g/L D(+) glucose and 17‐g/L agar) (Merck Millipore Brasil) at 30°C for 24 h (TE‐371/240L Incubator, Tecnal Equipamentos Científicos Brasil). After growth, the fungi were stored at 4°C and transferred to a fresh medium each 3 months.

### 2.3. Larvae of the Insect *G*. *mellonella*

*G*. *mellonella* (greater wax moth, Lepidoptera: Pyralidae) larvae were reared in ventilated plastic containers with ad libitum artificial diet (250‐g cornmeal, 250‐g honey, 210‐g glycerol, 210‐g beeswax, 150‐g brewer′s yeast, 100‐g soy flour, and 100‐g skim milk powder). Eggs were placed under fresh diet and incubated at 27°C in the dark (TE‐371/240L incubator, Tecnal Equipamentos Científicos, Brazil). Diet was refreshed and containers cleaned as needed until pupation. For adult emergence, 10 pupae were moved to new ventilated containers without food. Kraft paper was supplied for oviposition and removed after 1–3 weeks. Collected eggs started subsequent generations. Experimental subjects were randomly selected consisting of final‐instar larvae (2–3 cm, approximately 0.275 g), exhibiting active movement and no visible melanization foci, and used immediately.

### 2.4. Determination of the Lethal Dose of Bioinspired Peptides on Fungi

Fungi were grown on Sabouraud agar at 30°C for 24 h. A colony was resuspended in 1‐mL Sabouraud broth (5 g/L of meat peptone, 5 g/L of casein peptone, 20‐g/L D(+) glucose) (Merck Millipore Brazil), and cell density was determined by direct counting in a Neubauer chamber (LaborOptik, United Kingdon) using the Axio Imager.A2 optical microscope (Zeiss) to calculate cells per milliliter.

The antifungal assay was performed according to Toledo et al. [16] and Wiegand et al. [20]. The assay was set up in a 96‐well polystyrene U‐bottom plate (Nunc, Thermo Scientific Brasil). Each well contained 2 × 10^3^ cells/mL, test peptide at varying concentrations, and Sabouraud broth to 100‐*μ*L final volume. Control contained only 2 × 10^3^ cells/mL and medium, totaling 100 *μ*L. Plate was incubated at 30°C for 6 h for RR, 3 h for D‐RR, and 1 h for WR [12] (TE‐371/240L Incubator, Tecnal Equipamentos Científicos, Brazil). After incubation, the content of each well was washed with 900 *μ*L of Sabouraud broth, centrifuged (5000xG) for 1 min, then, 900 *μ*L of supernatant was removed, and the cells were resuspended in the remaining 100 *μ*L, plated on Sabouraud agar using a Drigalski loop, and incubated at 30°C for 24 h before colony forming units (CFU) were counted. The LD_100_ was defined as the concentration (*μ*M) that produces complete loss of fungal viability. Viability was determined by the fungus′ ability to divide and form colonies under appropriate growth conditions, in the absence of peptide, after 24 h of incubation.

### 2.5. Determination of the Fungal Lethal Dose in *G. mellonella*

Aliquots from 24‐h fungal cultures were suspended in 1 mL of insect physiologic saline (IPS) (30 mM NaCl, 5 mM KCl, 100 mM TRIS‐HCl, 10 mM EDTA, 30‐mM sodium citrate, pH 6.9) [21] or phosphate buffered saline (PBS) (10 mM Na_2_HPO_4_, 1.8 mM KH_2_PO_4_, 137 mM NaCl, 2.7 mM KCl, pH 7.4). Cell density (cells/mL) was determined as described in Item 2.4. From the stock suspensions, inocula were prepared at 0.1 × 10^6^, 1.0 × 10^6^, and 10 × 10^6^ cells/mL for *C. tropicalis* ATCC 750, and 0.5 × 10^6^, 1.0 × 10^6^, and 2.0 × 10^6^ cells/mL for *C. albicans* SC 5314. The cuticle around the last left proleg was cleaned with 70% ethanol, and 10 *μ*L of each fungal inoculum was injected intrahemocoelically using an insulin syringe. Control larvae received only 10 *μ*L IPS or PBS. Inoculated larvae were kept in Petri dishes at 37°C (TE‐392/170L incubator, Tecnal Equipamentos Científicos, Brazil) and monitored every 24 h for 144 h to determine the survival probability and the health index (HI). The HI was determined according to the categories of Table 1. The “motility” category measured the motility of the larvae and their response to touch with tweezers. The “cocoon formation” category measured the cocoon formation capacity of the larvae; after assessment, cocoons were removed. The “melanization” category measured melanin production by visual analysis following the reference shown in Table 1 [22–26]. The “survival” category measured the survival of the larvae according to the progression of the fungal infection. Larvae death was defined as body melanization over 90% and lack of movement upon stimulation by tweezers. The lethal fungal dose was the concentration that leads to the gradual death of all larvae by 144 h after infection. Higher HI indicates better health.

**Table 1:** Health index scores parameters used to define larvae scores during experiments.

Category	Description	Score	Visual parameter	Maximum
Activity	Without movement	0	‐	3
	Minimal movement when stimulated	1		
	Move when stimulated	2		
	Move without stimuli	3		

Cocoon formation	Without	0	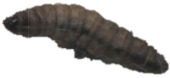	1
	Partial	0,5	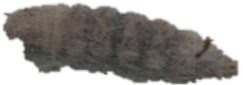	
	Total	1	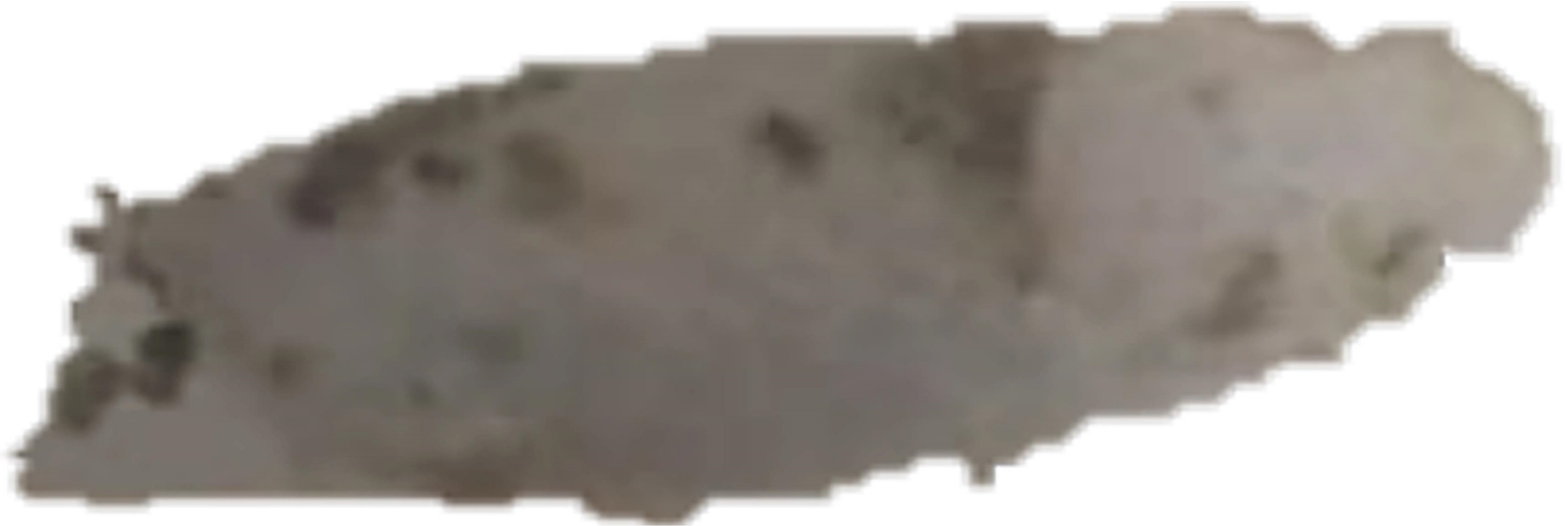	

Melanization	Black	0	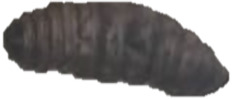	4
	> 40% body′s	1	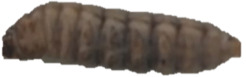	
	20%–40% body′s	2	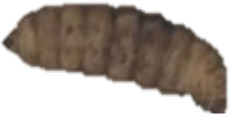	
	< 20% body′s	3	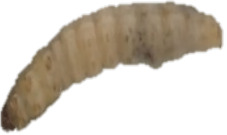	
	Without	4	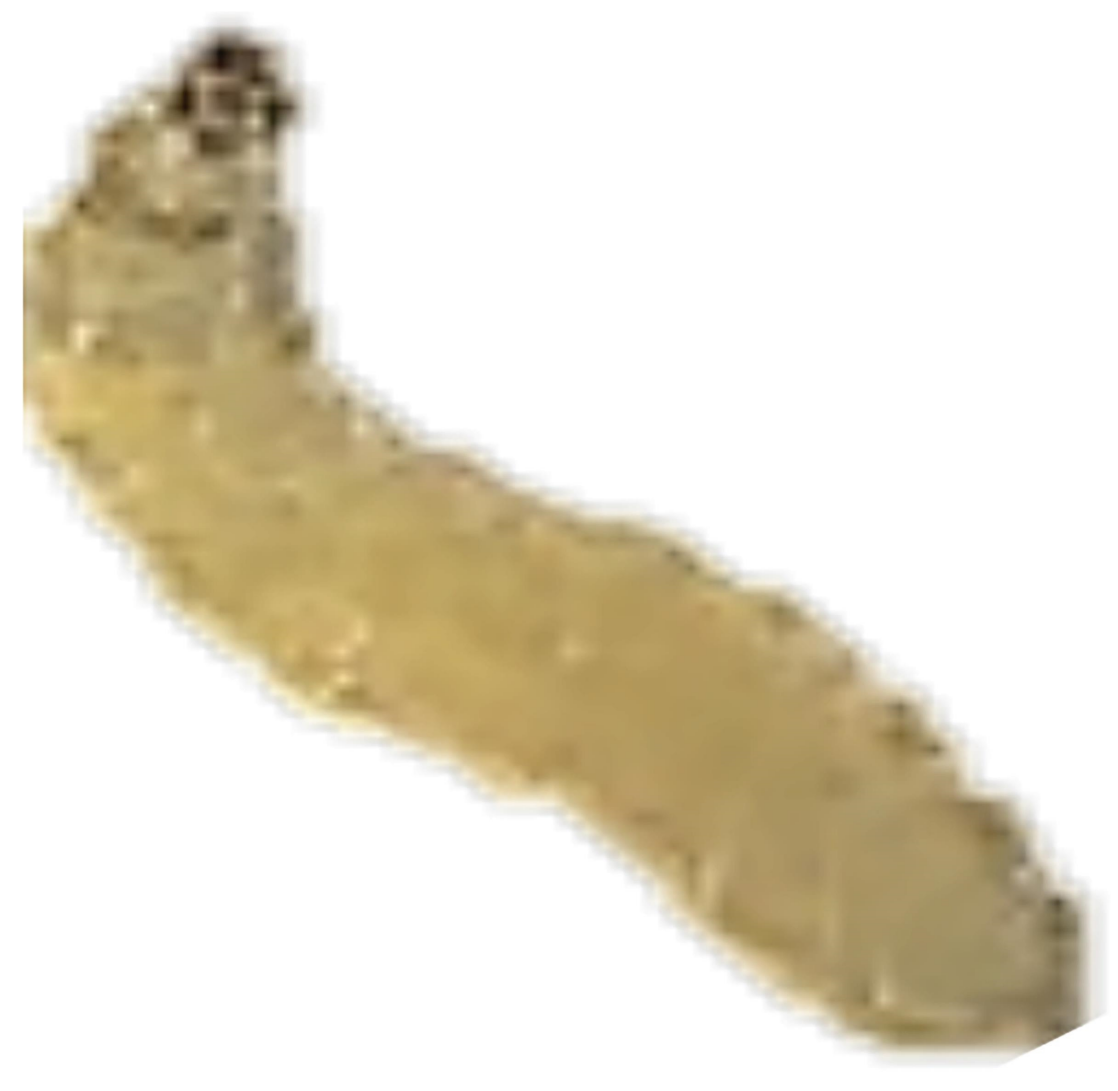	

Survival	Dead	0	‐	2
	Alive	2		

	Total			10

### 2.6. Determination of the Antifungal Activity of Bioinspired Peptides Against the Fungal Lethal Dose in *G. mellonella* Under In Vitro Conditions

Following FLD_100_ determination in *G. mellonella*, RR, D‐RR, and WR were tested in vitro against fungal inocula adjusted to the FLD_100_ cell density. The antifungal assay and viability followed the guidelines in Item 2.4 with two key modifications: inclusion of a Sabouraud‐broth blank to correct for medium absorbance, and use of the FLD_100_ cell density in control and peptide wells. Plates were read at 620 nm (EZ Read 400, Biochrom) immediately after setup (0 h) to record baseline turbidity. Samples were incubated at 30°C for each peptide′s lethal exposure time, 6 h for RR, 3 h for D‐RR, and 1 h for WR, based on Toledo et al. [16]. After incubation, absorbance at 620 nm was measured and percentage inhibition was calculated relative to untreated controls.

### 2.7. Determination of Fungal Load in *G. mellonella* Treated With the Bioinspired Peptides

Five larvae with average HI were chosen. The ventral surface of each larva was disinfected with 70% ethanol, and the larva′s right proleg punctured with a sterile insulin syringe to collect 50‐*μ*L hemolymph. Hemolymph samples were taken at 4, 24, and 48 h postinfection and transferred to a prechilled microcentrifuge tube containing an equal volume of tropolone solution (0.01 g/mL) to inhibit polyphenol oxidase cascade. Aliquots were diluted in Sabouraud broth, 1:10 (*V*/*V*) for *C. albicans* SC 5314 and 1:100 (*V*/*V*) for the *C. tropicalis* ATCC 750, plated on Sabouraud agar supplemented with ampicillin (50 *μ*g/mL) and chloramphenicol (34 *μ*g/mL), spread with a Drigalski loop and incubated at 30°C for 24 h. CFU were counted, and the fungal load (CFU/mL) was calculated as CFU/mL = (CFU × 1st dilution in tropolone × 2nd dilution in Sabouraud × 1000 *μ*L)/50 *μ*L.

### 2.8. Assessment of Hemocyte Concentrations in the Hemolymph During the Progression of the Infection

Because *G. mellonella* innate immunity parallels mammals [27], we measured the cellular response to lethal fungal infection. Hemolymph was harvested at 4, 24, and 48 h postinfection (and from controls) as described in Item 2.7, and immediately diluted 1:1 (*V*/*V*) in a 0.01‐g/mL tropolone solution to inhibit phenoloxidase cascade activity, then further diluted 1:10 (*V*/*V*) in IPS. A 10‐*μ*L aliquot of each dilution was loaded into a Neubauer chamber (LaborOptik, United Kingdom) and examined by optical microscope (Axio Imager.A2, Zeiss) to enumerate hemocytes. Hemocyte density was calculated as Cell/mL = (number of cells × 1st dilution in tropolone × 2nd dilution in IPS × 1000 *μ*L)/50 *μ*L.

### 2.9. In Vivo Efficacy and Toxicity of Bioinspired Peptides on Model Fungal Infection in *G. mellonella* Larvae

Lethal doses of *C. tropicalis* ATCC 750 and *C. albicans* SC 5314 for *G. mellonella* larvae were prepared according to the Methods 2.5. Peptides concentrations were based on each peptide′s LD_100_ and prepared as described in Item 2.1. Injection times for the peptides into the last right proleg of the larvae were chosen based on three criteria: fungal load progression, hemocyte concentration changes during infection, and the survival probability curve. Peptide doses were calculated assuming 2‐mg/mL concentration (in water), > 95% purity, and an average larval weight of 0.275 g. Final doses were 30 mg/kg for RR, 22 mg/kg for D‐RR, and 40 mg/kg for WR. Fluconazole (FCZ) was used as comparator at 400 mg/kg based on prescription guidelines [28]. The injections schedules were as follows: RR and D‐RR, four doses were injected at 6, 12, 24, and 96 h postinfection, and WR three doses at 8, 32, and 56 h postinfection. Controls (PBS or IPS) and FCZ were injected on the same schedule to match volume and needle injury. After injections, larvae were kept at 37°C for up to 144 h, with HI evaluated daily.

### 2.10. In Vitro Antifungal Assay Under Mammalian‐Like Physiological Conditions

The in vitro antifungal assay was also performed in Roswell Park Memorial Institute (RPMI) 1640 medium (Sigma‐Aldrich, R6504) to simulate mammalian physiological conditions, using the lethal dose of each peptide–fungus combination that was previously determined in Sabouraud medium. The assay followed the procedure described in Item 2.4, with the following modifications: RPMI 1640 was prepared according to the considerations of Boone et al. [29], with the glucose concentration adjusted to 4% to match that of Sabouraud broth. The pH was adjusted to 7.4 using 165 mM 3‐(N‐morpholino) propanesulfonic acid (MOPS, Sigma‐Aldrich), and the assay was incubated at 30°C. Additionally, we performed the antifungal assay in RPMI 1640, where MOPS was replaced with 23‐mM sodium bicarbonate (Sigma‐Aldrich), adjusted to pH 7.4, following the guidelines of Dorschner et al. [30], Belanger et al. [31], Nizet [32], and Ersoy et al. [33]. In this case, the assay was incubated at 37°C.

### 2.11. Larval Dissection

Five larvae per treatment group (as defined in Item 2.9) were frozen at −4°C for at least 24 h, then ventrally opened with a No. 24 scalpel blade, and placed in a Petri dish. Internal organs were exposed with fine forceps, and examined under a binocular stereomicroscope (SMZ 160T, Motic) equipped with an illumination system (Jenalux 20) to assess nodulation, melanization, and organ integrity. High‐resolution images (4619 × 3464 pixels) were captured using a mobile device equipped with a 16 MP mobile camera (Moto G8 Power Lite XT2055, XT2055‐2) [34, 35].

### 2.12. Posttreatment Evaluation Beyond 144 h

To evaluate the long‐term impact of WR treatment, 12 larvae were randomly chosen after 144 h and monitored for another 8 weeks to track their development into the adult moths. The specimens were reintroduced to an artificial diet recorded weekly at 7‐day intervals during the observation period.

### 2.13. Statistical Analysis

**Figure 1:**
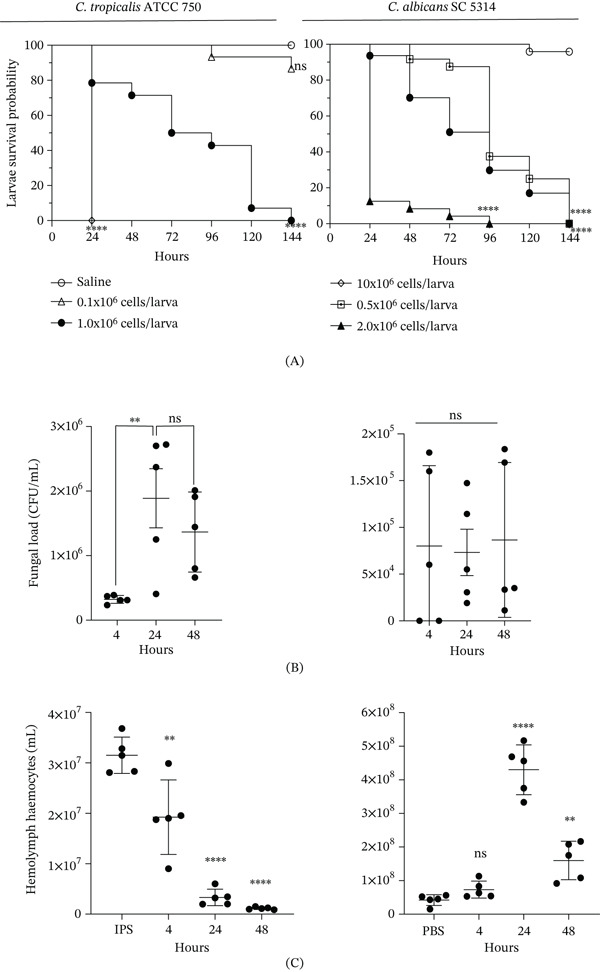
Infection parameters of *Galleria mellonella* larvae exposed to varying inoculum concentrations of clinical fungal strains. For both fungi: (A) Kaplan–Meier survival probability curves over a 144 h (6‐day) period revealed statistically significant differences among all groups. Each experiment included 15 larvae, with saline (PBS or IPS) as the control. Results represent the mean of three independent biological replicates (*n* = 180 total). (B) Fungal load of infected larvae, with each point representing an individual measurement. Results represent the mean of one independent biological replicate with *n* = 5 for each timepoint. (C) Hemocyte density in larvae infected with FLD_100_ across different timepoints, shown as an individual data point. Results represent the mean of one independent biological replicate with *n* = 5 for each timepoint. Standard deviations were statistically different. Hemocyte number in *G. mellonella* injected with IPS or PBS did present a statistical difference (Figure S2).

**Figure 2:**
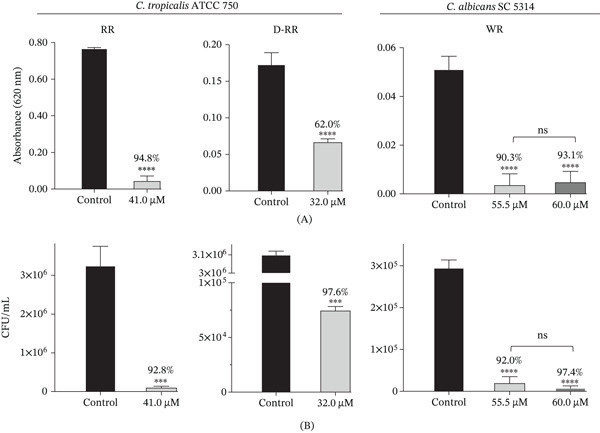
Lethal dose assessments of bioinspired peptides against clinical fungal strains. (A) Growth inhibition for each peptide against its corresponding fungal strain at FLD_100_. (B) Lethal dose of each peptide against FLD_100_ in *G. mellonella* larvae. Results represent the mean of three independent biological replicates, each consisting of three samples (n = 9 total).

**Figure 3:**
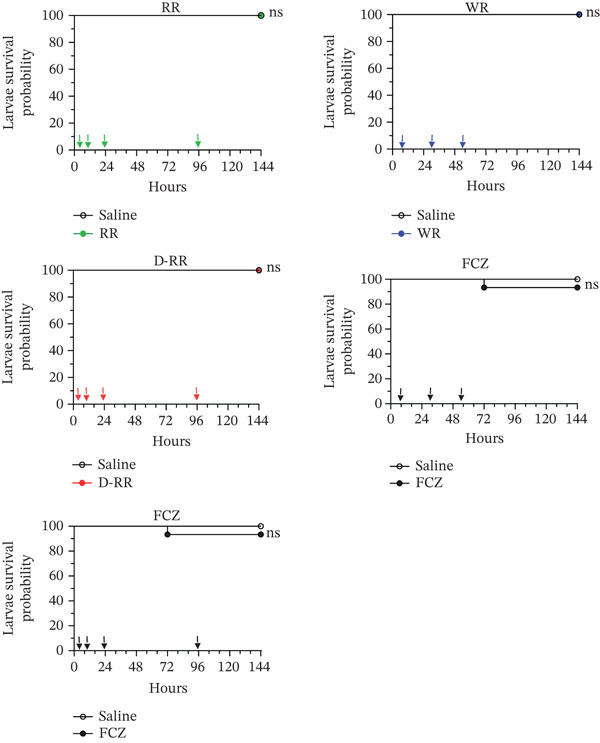
Kaplan–Meier survival curves from toxicity assay in *Galleria mellonella* larvae monitored over 144 h (6 days). Survival curves for saline, RR, D‐RR, and WR treatments showed no statistically significant differences. Likewise, no significant difference in survival rates was observed between larvae receiving three or four doses of FCZ. Colored arrows indicate the timing and number of administrations of saline, peptides, or FCZ. Results represent the mean of three independent biological replicates, each consisting of 5 larvae per sample (*n* = 105 total).

**Figure 4:**
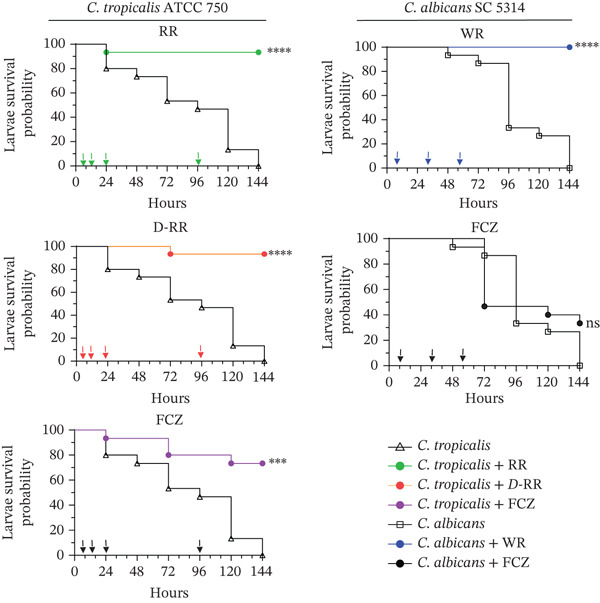
Kaplan–Meier survival curves of efficacy tests of *Galleria mellonella* larvae under different treatments over 144 h (6 days). The peptides RR, D‐RR, and WR significantly improved survival compared with untreated controls (*p* < 0.0001), with no statistically significant (ns) differences observed among peptide‐treated groups (Table S5). Fluconazole (FCZ), used as a reference antifungal, was effective only for the *Candida tropicalis* infection (*p* < 0.001), with RR and D‐RR showing comparable efficacy (ns). WR demonstrated superior effectiveness against *Candida albicans*, significantly outperforming FCZ (*p* = 0.0001). Colored arrows on the survival curves indicate the timing and number of treatment administrations. Results represent the mean of three independent biological replicates, each consisting of five larvae per sample (*n* = 105 total).

**Figure 5:**
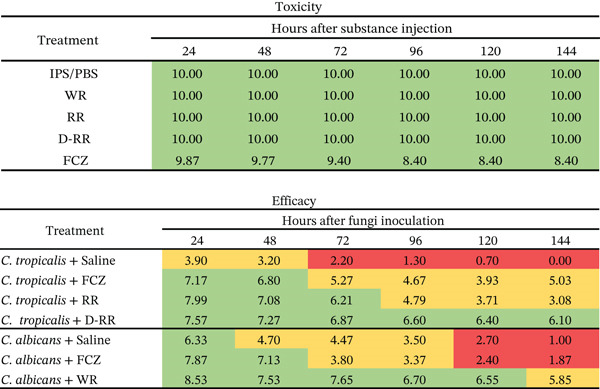
(A) Toxicity and (B) efficacy of the bioinspired peptides were assessed in *Galleria mellonella* larvae using health index (HI) scoring. (A) In toxicity assays, larvae were injected with saline at 0 h, followed by either saline or peptide treatment according to the designated schedule. Fluconazole (FCZ) served as a reference, and it was the only treatment that showed a statistically significant difference compared with the others, and no time‐dependent effects were observed. Results represent the mean of three independent biological replicates, each consisting of 15 larvae per sample (*n* = 180 total). (B) In efficacy assays, control groups received saline injections at matched timepoints to simulate needle injury and to ensure equivalent injection volumes. FCZ was administered following the same schedule. Treatment with the peptides produced significant, time‐dependent effects between 48 and 96 h compared with the respective controls. Larval health was color coded: green for HI > 60*%*, yellow for HI between 30% and 60%, and red for HI < 30*%*. Results represent the mean of three independent biological replicates, each consisting of five larvae per sample (*n* = 105 total).

**Figure 6:**
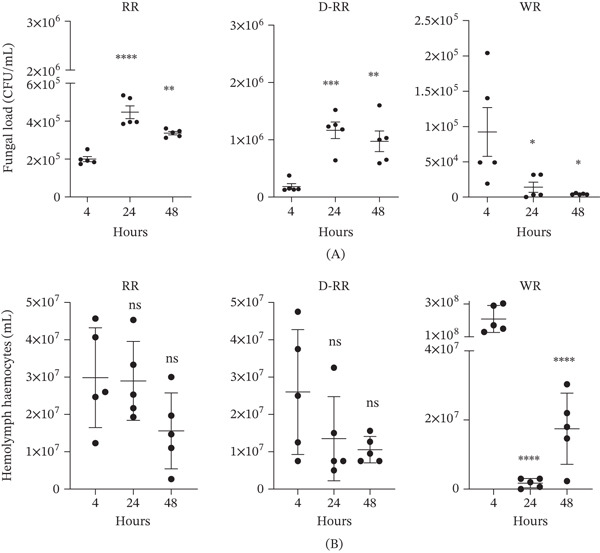
(A) Fungal burden in peptide‐treated *Galleria mellonella* larvae, with each point representing an individual data value. Results represent the mean of one independent biological replicate with *n* = 5 for each timepoint. (B) Hemocyte density at various timepoints in peptide‐treated larvae, shown as individual data points. Results represent the mean of one independent biological replicate with *n* = 5 for each timepoint.

**Figure 7:**
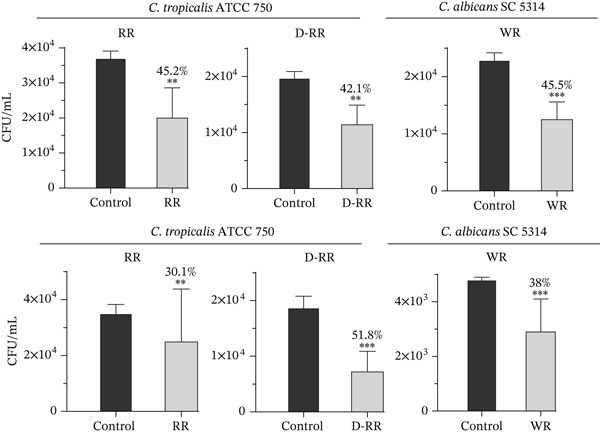
Antifungal assay of peptides in RPMI 1640 medium. (A) Antifungal assay performed in RPMI 1640 medium supplemented with metal ions, MOPS, and glucose. (B) Antifungal assay performed in RPMI 1640 medium supplemented with sodium bicarbonate and incubated at 37°C for best mimicry of the mammalian host‐like conditions. The percentage of viability loss is indicated above each test bar. *n* = 9 for each timepoint.

All assays were conducted using three independent biological replicates, unless otherwise stated, as indicated in the corresponding figure legends. Statistical analyses were as follows: in Figure 1, (A) Gehan–Breslow–Wilcoxon test, (B) ordinary one‐way ANOVA Turkey test, and (C) Bartlett test; in Figure 2, (A) and (B), unpaired *t*‐test statistical significance was compared with controls; in Figure 3, Gehan–Breslow–Wilcoxon and Mantel–Cox tests; in Figure 4, Gehan–Breslow–Wilcoxon and Mantel–Cox tests (*p* values are shown in Table S5); in Figure 5, (A) two‐way ANOVA and Tukey multiple comparisons test, *p* < 0.001, and (B) two‐way ANOVA and Tukey multiple comparisons test, *p* < 0.0001; in Figure 6, (A) ordinary one‐way ANOVA Turkey test; and in Figure 7 (A and B), unpaired *t*‐test. Significance levels are as follows: (∗) *p* < 0.1, (∗∗) *p* < 0.01, (∗∗∗) *p* < 0.001, and (∗∗∗∗) *p* < 0.0001; (ns) not significant. All statistical evaluations were carried out using GraphPad Prism Version 8.0.2 (GraphPad Software, Inc., United States). Data are presented as mean ± standard deviation.

## 3. Results

### 3.1. Fungal Lethal Dose of *C. tropicalis* ATCC 750 and *C. albicans* SC 5314 in *G. mellonella* Larvae

We determined the fungal lethal dose (FLD_100_) of each *Candida* species in *G. mellonella* larvae by Kaplan–Meier survival analysis. *C. tropicalis* ATCC 750 exhibited a dose‐dependent decrease in survival: 0.1 × 10^6^ cells/larva yield 93% survival at 144 h, 1 × 10^6^cells/larva resulted in 0% survival at 144 h, and 10 × 10^6^cells/larva produced 0% survival within 24 h (Figure 1A). For the *C. albicans* SC 5314, it also showed load‐dependent lethality: 0.5 × 10^6^cells/larva gave 0% survival at 144 h, 1 × 10^6^cells/larva also produced 0% survival at 144 h, and 2 × 10^6^cells/larva resulted in 0% survival by 96 h (Figure 1A). Saline‐injected controls retained 100% survival through 144 h for both fungal species (Figure 1A).

In parallel, HI scoring over 144 h mirrored survival outcomes. For *C. tropicalis* ATCC 750, HI declines dose‐dependently: 0.1 × 10^6^cells/larva dropped from 9.3 at 24 h to 7.7 at 144 h, 1 × 10^6^cells/larva fell from 3.9 at 24 h to 0.0 at 144 h, and 10 × 10^6^cells/larva reached HI 0.0 by 24 h (Table S1). For *C. albicans* SC 5314: 0.5 × 10^6^cells/larva reduced HI from 8.37 at the 24 h to 0.04 at 144 h, 1.0 × 10^6^cells/larva dropped HI from 6.05 at 24 h to 0.0 at 144 h, and 2.0 × 10^6^cells/larva produced an HI 0.0 with complete mortality by 96 h (Table S1). Saline controls maintained high HI throughout (Table S1). Individual HI subcategories mirrored these overall declines (Table S2). Using Kaplan–Meier survival curve and HI, we selected 1.0 × 10^6^cells/larvafor *C. tropicalis* ATCC 750 and 0.5 × 10^6^cells/larvafor *C. albicans* SC 5314 since they produced gradual disease progression culminating in 100% mortality by 144 h, therefore, defining the FLD_100_ (Figure 1A).

At the selected FLD_100_, we measured hemolymph fungal burden and circulating hemocytes at 4, 24, and 48 h. *C. tropicalis* ATCC 750, colony counts rose from 3.22 × 10^5^ CFU/mL at 4 h to 1.89 × 10^6^ CFU/mL at 24 h, corresponding to roughly a six‐fold expansion (0.77 log_10_), then declined slightly to 1.37 × 10^6^ CFU/mL by 48 h, approximately 1.4‐fold reduction (0.14 log_10_) (Figure 1B). Observation of numerous pseudohyphae in the hemolymph at 24 h corroborated the advanced stage of infection (result not shown). In contrast, *C. albicans* SC 5314 maintained a stable fungal load of approximately 8.00 × 10^4^ CFU/mL throughout the 48‐h interval (Figure 1B). Parallel assessment of host immunity revealed distinct hemocyte dynamics for each species. In *C. tropicalis* ATCC 750–infected larvae, circulating hemocytes fell from 3.15 × 10^7^ hemocytes/mL in uninfected controls to 1.92 × 10^6^ hemocytes/mL at 4 h, fell modestly at 3.33 × 10^6^ hemocytes/mL by 24 h, and then dropped to 1.15 × 10^6^ hemocytes/mL at 48 h. By comparison, *C. albicans* SC 5314 infection induced a pronounced hemocytes surge, reaching 4.30 × 10^8^ hemocytes/mL at 24 h before declining to 1.60 × 10^8^ hemocytes/mL at 48 h, highlighting this time interval as critical phase of immune activation (Figure 1C). These kinetics confirm successful establishment of the fungal infection in the tissue milieu of *G. mellonella* and the activation of immune cell response, and delineate an optimal therapeutic window for antifungal peptide administration. Based on the simultaneous rise in fungal burden and hemocyte response, we propose initiating peptide treatment between 4 h, when host defenses begin to mobilize, and 96 h postinfection to maximize efficacy against both *Candida* species.

### 3.2. Determination of the Lethal Dose of Bioinspired Peptides In Vitro on *Candida* spp.

We initially assessed whether the previously established LD_100_ values, 27.5 *μ*M for RR and 23 *μ*M for D‐RR against *C. tropicalis* C017, and 27.5 *μ*M of WR against *C. albicans* C022, were applicable to clinical isolates at a cell density of 2 × 10^3^ cells/mL^16^. To ensure methodological consistency and enable direct comparison with the previously tested strains, we used the same culture medium, Sabouraud, as in earlier studies. Under these conditions, none of the original concentrations achieved complete lethality (Table S3). Consequently, peptide concentrations were increased in 4.5‐*μ*M increments until full cell death was observed. RR eradicated *C. tropicalis* ATCC 750 at 41 *μ*M, whereas D‐RR reached LD_100_ at 32 *μ*M. For *C. albicans* SC 5314, WR required 60 *μ*M to achieve 99.5% reduction in viability. These values define the LD_100_ for the clinical strains under our assay conditions (Table S3). Variations in CFU relative to controls are attributed to differences in peptide incubation times [16].

### 3.3. Evaluation of the Antifungal Activity of RR, D‐RR, and WR Against the Lethal Fungal Dose of *Candida* Spp. in *G. mellonella* Under In Vitro Conditions

Each peptide′s LD_100_ was confirmed at 2 × 10^3^ cells/mL (Table S3), and then tested against the previously determined FLD_100_ of each fungal species in G. *mellonella* larvae (Figure 1). At an FLD_100_ of 1.0 × 10^6^ C. *tropicalis* ATCC 750 cells/larva, RR at 41 *μ*M produced 98.2% killing, and D‐RR at 32 *μ*M produced 97.6% killing. At an FLD_100_ of 0.5 × 10^6^ C. *albicans* SC 5314 cells/larva, WR at 60 *μ*M produced 97.4% lethality (Figure 2). A concentration of 55.5 *μ*M of WR was also tested with the aim of minimizing peptide usage during treatment and this concentration achieved 92% kill.

These results define the operational lethal doses for each peptide‐fungus pair under the assay conditions and support using RR 41 *μ*M, D‐RR 32 *μ*M, and WR 60 *μ*M for in vivo efficacy testing (55.5 *μ*M WR noted as a lower efficacy alternative).

### 3.4. In Vivo Assessment of the Toxicity and Efficacy of Treatments Using Bioinspired Peptides

We evaluated in vivo toxicity of the peptides in *G. mellonella* after establishing FLD_100_ and peptide LD_100_ values. Treatment timing was based on survival date, peak fungal burden at 24 h, and hemocyte declines at 48 h. RR (30 mg/kg) and D‐RR (22 mg/kg) were administered at 6, 12, 24, and 96 h postinfection (arrows). WR (40 mg/kg) was administered at 6, 32, and 56 h (arrows). Survival in all peptide‐treated groups was comparable to saline‐injected controls, confirming no detectable toxicity at those doses (Figure 3). By contrast, the reference drug FCZ, given at 400 mg/kg [18] on the same RR and D‐RR schedule (four injections, arrows), and WR schedule (three injections, arrows), caused some toxicity, with 93.3% survival at 72 h postinjection (Figure 3). To further confirm the absence of peptide toxicity in *G. mellonella* larvae, parameters including motility, melanization, and cocoon formation were monitored over a 144‐h period. None of these parameters exhibited any alterations, indicating that the peptides were nontoxic (Table S4).

Peptide efficacy was evaluated in *G. mellonella* infected at the previously determined FLD_100_, *C. tropicalis* ATCC 750 (1.0 × 10^6^ cells/larva) or *C. albicans* SC 5314 (0.5 × 10^6^ cells/larva), using optimized, nontoxic dosing regimens established in the toxicity assay. Peptide doses applied in vivo corresponded to the in vitro lethal concentrations (Figure 2). Untreated infected larvae exhibited progressive mortality, reaching 0% survival by 144 h for both fungi (Figure 4). In contrast, RR and D‐RR treatments delivered postinfection conferred 93.3% survival at 144 h against *C. tropicalis* ATCC 750, and WR treatment produced 100% survival in *C. albicans* SC 5314‐infected larvae (Figure 4). FCZ (400 mg/kg) offered only transient protection: in *C. tropicalis* ATCC 750 infections, survival declined from 93.3% at 24 h to 73.3% at 144 h, and in *C. albicans* SC 5314 infections, survival dropped from 100% at 24 h to 43.3% at 72 h (coinciding with treatment cessation) and further to 33.3% at 144 h (Figure 4). The fungi were inoculated in PBS or IPS and the infection was allowed to establish. Consequently, *C. tropicalis* and *C. albicans* adapted to the tissue milieu of *G. mellonella* when the peptides, diluted in water, were administered to the larvae (Figure 3). Therefore, the observed inhibitory effects of the peptides occurred in the in vivo tissue environment of *G. mellonella*. Combining the in vitro inhibitory effects shown in Figure 2 with the in vivo activity presented in Figure 3 demonstrates that the peptides retain inhibitory activity under both experimental conditions.

HI measurements corroborated the survival data (Figure 5B and Table S6). PBS‐injected larvae exhibited a gradual decline in the HI from 72 h onward, reaching a score of 0 by 144 h, indicating uncontrolled progression of the infection. Larvae treated with RR, D‐RR, or WR maintained stable HI scores over 144 h, indicating preservation of physiological status. By contrast, FCZ‐treatment produced a HI marked decline beginning at 72 h postinfection for both fungi. Peptide‐treated groups retained significantly higher HI values than the FCZ group, confirming lower toxicity and superior maintenance of larval health. Among the peptides, WR showed the strongest overall benefit on HI, supporting its potential as a lead antifungal candidate.

Fungal burden and circulating hemocyte were quantified in *G. mellonella* following peptide treatment using previously established methods (Figure 6). In RR‐treated larvae, fungal load increased from 2.0 × 10^5^ CFU/mL at 4 h to 4.5 × 10^5^ CFU/mL at 24 h, approximately 2.2‐fold increase (0.35 log_10_), following a decrease to 3.4 × 10^5^ CFU/mL at 48 h, approximately 1.3‐fold reduction (0.12 log_10_). For D‐RR–treated larvae, fungal load rising from 1.8 × 10^5^ CFU/mL at 4 h to 1.2 × 10^6^ CFU/mL at 24 h representing an approximately 6.7‐fold expansion (0.85 log_10_), then decreasing to 9.7 × 10^5^ CFU/mL at 48 h, approximately 1.2‐fold reduction (0.09 log_10_). WR‐treated larvae fungal load declined from 9.2 × 10^4^ CFU/mL at 4 h to 1.4 × 10^4^ CFU/mL at 24 h, corresponding to approximately 6.6 reduction (0.82 log_10_), and reaching 4.2 × 10^3^ CFU/mL at 48 h, representing an additional 3.4 reduction (0.52 log_10_). No statistically significant changes in hemocyte density were observed over time in for RR‐ and D‐RR–treated larvae, despite slight reduction in mean values. However, WR treatment resulted in a marked decline in hemocyte density, decreasing from 2.1 × 10^8^ cells/mL at 4 h to 1.7 × 10^6^ cells/mL at 24 h and 1.7 × 10^7^ cells/mL at 48 h. Summary interpretation: RR and D‐RR allowed transient fungal growth peaking at 24 h with subsequent partial reduction and no significant hemocyte depletion. WR produced the greatest early reduction in fungal load but was associated with a substantial, time‐dependent drop in hemocyte counts.

### 3.5. In Vitro Antifungal Assay Under Mammalian‐Like Physiological Conditions

At the LD_100_ of the peptides determined in Sabouraud broth, the antifungal assay was performed against clinical fungal strains in RPMI 1640 medium supplemented with metal ions, MOPS, and glucose [29] (Table S3). Under these conditions, *C*. *tropicalis* ATCC 750 showed a viability loss of 45.2% when incubated with RR and 42.1% with D‐RR, whereas *C*. *albicans* SC5314 exhibited a 45.5% viability loss (Figure 7A). We also evaluated peptide antifungal activity in RPMI 1640 medium supplemented with sodium bicarbonate and incubated at 37°C. In this condition, viability loss was 30.1% and 51.8% for *C. tropicalis* ATCC 750 incubated with RR and D‐RR, respectively, and 38% for *C. albicans* SC5314 incubated with WR (Figure 7B). In both conditions, viability loss did not reach 100% as observed in Sabouraud broth, because peptide concentrations were not adjusted for RPMI 1640 medium as they were for Sabouraud (Table S3).

### 3.6. Larval Dissection

**Figure 8:**
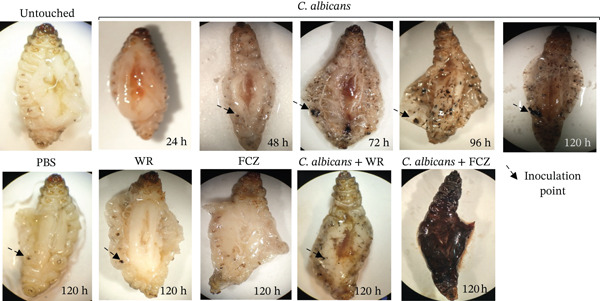
Dissections of *Galleria mellonela* larvae following treatment regimens. Nodule formation was observed in all larvae infected with *C. albicans* SC 5314, with increasing frequency and severity over time. Larvae treated with the WR peptide exhibited reduced internal organ damage compared with infected controls. Both WR and fluconazole (FCZ) treatments were nontoxic, with internal morphology resembling that of untouched and PBS‐injected larvae. However, FCZ failed to effectively control the infection. Melanization caused by injection injury (indicated by arrows) was excluded from comparative analysis. Results represent the mean of one independent biological replicate with *n* = 5.

Given WR′s superior efficacy in survival efficacy, we performed time‐course dissections to evaluate melanization, nodule formation, and internal organ integrity. In untreated larvae infected with *C. albicans* SC 5314 at the FLD_100_, infection progressed steadily: by 120 h, the fat body and intestine exhibited substantial degradation, and extensive nodule formation (Figure 8). Larvae treated with WR alone were indistinguishable from PBS controls, with no melanization, nodulation, or organ damage (Figure 8), confirming WR′s nontoxicity (Figure 3). When WR was administered to infected larvae, melanization and tissue invasion remained minimal at 120 h, comparable to the 72‐h stage of untreated larvae, indicating effective containment of fungal progression (Figure 8). FCZ, despite being nontoxic under the same regimen (Figure 3), did not prevent extensive organ damage by 120 h (Figure 8).

### 3.7. Posttreatment Evaluation Beyond 144 H

**Figure 9:**
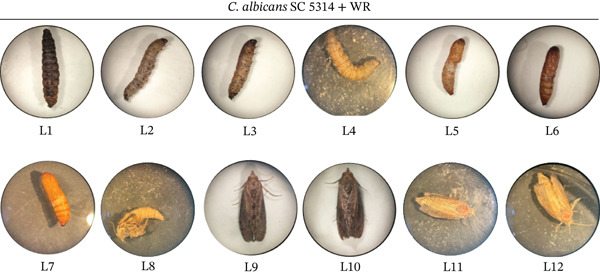
Posttreatment evaluation of *Galleria mellonella* larvae following WR administration was conducted 4 weeks after efficacy testing. Images show developmental outcomes of 12 larvae (L1–L12). Four successfully reached adulthood with normal morphology, intact antennae and legs, no melanization, and full flight ability. The remaining larvae exhibited varying degrees of developmental impairment, including melanization, incomplete pupation, and failure to emerge from cocoons. Results represent one independent biological replicate (*n* = 12).

WR‐treated *G. mellonella* (*n* = 12) were followed for 8 weeks to evaluate long term effects on development and survivorship (Figure 9). Four larvae died during the larval stage (L1–L4). Of the eight larvae that metamorphosis, three (L5‐L7) died during prepupal or pupal stages, one (L8) arrested metamorphosis prior adult emergency. Four larvae (L9–L12) successfully completed metamorphosis to adults with no observable morphological abnormalities or developmental delay (Figure 9). These results indicate that WR treatment permits infection clearance while allowing a substantial proportion of larvae to complete normal life‐cycle progression.

## 4. Discussion

In prior investigations, Toledo et al. [16] and later confirmed by Lucas et al. [17] demonstrated the antifungal activity of the RR and D‐RR peptides against *C. tropicalis* CE017 and WR against *C. albicans* CE022. We re‐evaluated the efficacy of these peptides using the clinical isolates included in this study. Although Toledo et al. [16] reported complete cell death at the concentrations of 27.5 *μ*M RR, 23 *μ*M D‐RR, and 27.5 *μ*M WR, these doses did not produce similar mortability in our clinical strains under identical conditions. These variations may be explained because fungal cells deploy multiple strategies to evade AMP activity, including secretion of peptide effectors [36, 37], upregulation of peptide efflux pumps [38], and modulation of signaling pathways [39].

Protease secretion, particularly secreted aspartyl proteinases (Saps), has been documented as a key defense mechanism [40, 41]. Saps facilitate tissue invasion and colonization by disrupting host mucosal membranes and degrading essential immune and structural proteins, such as peptides with antimicrobial properties [40, 41]. In *C. tropicalis*, at least four SAP genes, SAPT1, SAPT2, SAPT3, and SAPT4, have been identified [42].

The virulence of the *C. albicans* SC 5314/ATCC MYA‐2876 is linked to heterozygosity at the regulator of biofilm 1 (ROB1) gene, which harbors the alleles ROB1^946P^ (ancestral) and ROB1^946S^ (rare). In oropharyngeal infections, the ROB1^946S^ allele facilitates mucosal penetration, whereas ROB1^946P^ favors a commensal phenotype. The ROB1^946S^ allele confers a gain‐of‐function effect enhancing filamentation, biofilm formation, and tissue invasion, thereby increasing pathogenicity. Among 223 *C. albicans* strains, SC 5314/ATCC MYA‐2876 is uniquely reported to exhibit this heterozygous profile and is considered one of the most invasive and filamentous. Despite genetic similarities with *C. tropicalis*, divergence at the ROB1 locus suggests that this strain has acquired a competitive advantage that enhances its virulence [43].

*G. mellonella* larvae have emerged as a viable in vivo alternative to murine models for evaluating the efficacy and toxicity of clinically relevant compounds [4, 23, 44–47, 48], pathogenesis [34, 49], fungal virulence [50], and implant associated infections [51].

An intermediate infection dose of 1.0 × 10^6^ cells/larva for *C. tropicalis* and 0.5 × 10^6^ cells/larva for *C. albicans* resulted in gradual disease progression with 100% mortality by 144 h (Figure 1 and Tables S1 and S2). Although survival rates did not differ significantly between doses, both maintained larva in a moribund state for an extended period, an advantageous condition for evaluating systemic candidiasis therapies. This prolonged morbidity mirrors clinical FCZ treatments for candidemia, which typically spans 7 days or more [28].

Fungal burden in the hemolymph is a key indicator of infection progression, correlating with prophenoloxidase activation and hemocyte levels. Higher fungal loads generally elicit stronger cellular immune responses. To assess fungal virulence and the *G. mellonella* immune response to FLD_100_, CFUs were quantified at defined intervals. For *C. tropicalis*, CFU levels peaked at 24 h postinfection, aligning with findings by Mesa‐Arango et al. [52], whereas hemocyte counts declined steadily. By contrast, *C. albicans* colonization was detectable as early as 4 h postinfection and remained stable. By 24 h, hemocyte levels peaked, indicating an acute immune response and suggesting possible tissue invasion [53–55]. The variation in hemocyte counts observed between infections with *C. tropicalis* ATCC 750 and *C. albicans* SC 5314 may reflect the distinct biological characteristics of each strain, such as differences in virulence and resistance profiles as in the ROB locus.

Following infection parameter characterized, we established a dosing regimen to evaluate the toxicity and efficacy of RR, D‐RR, and WR peptides. Standard FCZ therapy for systemic candidiasis involves multiple daily doses totaling 400 mg/kg/day, with a maximum of 600 mg/kg/day [28]. Based on these guidelines and our preliminary data (Figures 1 and 2 and Tables S1, S2, and S3), we derived equivalent doses of 30 mg/kg for RR, 22 mg/kg for D‐RR, and 40 mg/kg for WR. RR and D‐RR were administered at four timepoints: 4 h, in the stabilization of the fungal burden; 12 h, posthemocyte peak and the onset of tissue invasion; 24 h, early survival decline; and 96 h, approaching 50% mortality. WR was given three doses, following the same parameters, at 8, 32, and 56 h postinfection, omitting the final dose. This dose regimen effectively contained infection progression by both fungal species, demonstrating a favorable balance between toxicity and therapeutic efficacy.

Many AMPs display low toxicity toward mammalian cells, and synthetic optimization can further improve their potency, biocompatibility, and safety profiles [56]. To assess in vivo toxicity, RR, D‐RR, and WR were administered to *G. mellonella* larvae at the previously defined doses (Figure 2 and Table S3). No signs of toxicity were observable during the monitoring period (Figure 5 and Table S4). In the infected larvae treated according to our regimen, no mortality or behavioral abnormalities occurred (Figures 4 and 5 and Table S6). Surviving larvae complete metamorphosis without developmental delays or morphological defects, confirming the absence of sublethal toxicity (Figure 8). These in vivo findings aligned with Toledo et al. [16], who reported minimal toxicity of these peptides in murine RAW 264.7 macrophages and human THP‐1 monocytes. Similar safety profiles have been described for StigA8 and StigA18, analogs of the peptide Stigmurin, sharing similar physicochemical properties to RR, D‐RR, and WR, and showed no hemolytic activity or toxicity in *G. mellonella* [57].

Efficacy assays demonstrated that bioinspired peptides significantly improved survival of *Candida-*infected *G. mellonella* larvae. Survival rates in treated groups were comparable to saline‐injected controls and significantly higher than in untreated infected larvae (Figure 4). In addition to enhancing survival, peptide treatment improved overall larvae health, evidenced by lower melanization scores and increased HI (Figure 5 and Table S6). All peptides reduced fungal burden and hemocyte density, with WR showing the most pronounced effect (Figure 6).

The ability of the peptides to induce fungal viability loss in RPMI 1640 medium (Figure 7A,B) indicates that they remain active under conditions that mimic the mammalian environment [30–33]. Together with their demonstrated in vivo activity in *G. mellonella* (Figure 4), these findings highlight their therapeutic potential.

At 120 h postinfection, WR‐treated larvae exhibited markedly reduced melanization nodule formation (Figure 8). Visual assessment confirmed limited melanization compared with untreated, infected controls. Moreover, peptide‐treated larvae underwent normal metamorphosis without developmental delays or external morphological abnormalities, indicating preserved physiological integrity.

Despite larvae resuming development, growth was arrested at the prepupal or pupal stages. These metamorphic phases demand elevated metabolic output in lepidoptera [58], emphasizing the fat body′s critical role in fatty acid storage and systemic energy homeostasis. Phosphorylases activity in the fat body rises significantly during larval and pupal development to meet increased energy and glucose requirements, such as those required for chitin synthesis before pupation. This elevated activity continues through the pupal‐to‐adult transition, underscoring its metabolic relevance [59, 60]. Beyond metabolism, the fat body also plays key immune roles. In *Drosophila melanogaster*, it supports AMP production and counters infection‐induced oxidative stresses [60], whereas in *Ixodes ricinus*, it contributes to immune component synthesis [59]. As shown in Figure 7, *C. albicans* infection causes extensive tissue degradation, including severe damage to the fat body. These damages may be irreversible and, even with WR treatment, can compromise normal insect development.

Compared with the reference antifungal FCZ, peptides RR, D‐RR, and WR showed superior efficacy in enhancing larval survival and suppressing infection. Although FCZ treatment modestly improved the HI, its effect was significantly lower than that of the peptide. After treatment cessation, all groups exhibited a decline in HI, with the FCZ‐treated group showing the steepest drop. Data from Tables S4 and S6 and Figures 4 and 5B indicated that FCZ‐treated larvae were statistically indistinguishable from untreated, infected controls, highlighting the limited therapeutic benefit of the FCZ in this model.

Several synthetic peptides have shown strong antimicrobial activity. IKR18, a computationally designed peptide, and Epidermicin NI01, a synthetic derivative of epidermicin isolated from *Staphylococcus epidermidis*, effectively controlled infections by various strains of *Staphylococcus aureus* and *Acinetobacter baumannii* in the *G. mellonella* model [21]. Likewise, StigA8 and StigA18 provided in vivo protection against *S. aureus* in the *G. mellonella* model, with a single 40 mg/kg dose producing clear, dose‐dependent antimicrobial effects.

Within the study′s framework, peptides RR, D‐RR, and WR were shown to be nontoxic and effective in treating clinical *Candida* spp. infections in the *G. mellonella* model. Future investigations will aim to elucidate peptide degradation and metabolic pathways, assess effects on larval metabolism, exploring in situ peptide production, optimize administration routes, and evaluated efficacy and safety in mammalian models. These efforts will support the development of RR, D‐RR, and WR as promising pharmacological alternatives.

In conclusion, our study confirms that bioinspired peptides with antimicrobial properties (RR, D‐RR, and WR) are safe and highly effective antifungal candidates. At the tested doses (RR: 30 mg/kg, D‐RR: 22 mg/kg, and WR: 40 mg/kg), they showed no toxicity in *G. mellonella* larvae, with health outcomes comparable to saline controls. In contrast, the standard clinical antifungal FCZ (400 mg/kg) failed to match these outcomes, offering only transient protection. All peptides significantly improved larval survival against lethal *C. tropicalis* ATCC 750 or *C. albicans* SC 5314 infections. RR and D‐RR conferred 93.3% survival at 144 h against *C. tropicalis*, whereas WR achieved 100% survival in *C. albicans*‐infected larvae. WR stood out as the most potent candidate, preserving larval health and organ integrity, reducing melanization and nodule formation, and enabling full metamorphosis into healthy adults. These findings highlight RR, D‐RR, and especially WR, as promising antifungal candidates. Future work will investigate peptide degradation, host metabolism, in situ peptide production strategies, and optimizing administration routes, with mammalian trials as the next critical step.

**Nomenclature**
CFU: colony forming unit
FCZ: fluconazole
FLD: fungal lethal dose
HI: health index
IPS: insect physiologic saline
PBS: phosphate buffered saline

## Author Contributions

Alan da Silva Conceição: conceptualization, formal analysis, investigation, methodology, and writing—original draft preparation. Lorena Mendes dos Santos: conceptualization, formal analysis, investigation, and methodology. Gabriel Bonan Taveira: methodology. Valdirene Moreira Gomes: methodology and investigation. André de Oliveira Carvalho: conceptualization, formal analysis, investigation, methodology, writing—original draft preparation, project administration, supervision, writing—original draft preparation, and writing—review and editing. Allan da Silva Conceição and Lorena Mendes dos Santos contributed equally to this work.

## Funding

This study was supported by the Conselho Nacional de Desenvolvimento Científico e Tecnológico (10.13039/501100003593) (306429/2023‐3), Fundação Carlos Chagas Filho de Amparo à Pesquisa do Estado do Rio de Janeiro (10.13039/501100004586) (E‐26/210.484/2024‐APQ1; E‐26/203.948/2024‐Bolsa), Coordenação de Aperfeiçoamento de Pessoal de Nível Superior (10.13039/501100002322) (001), and Universidade Estadual do Norte Fluminense Darcy Ribeiro, PAPIC (SEI‐260002/007051/2024 ‐ AUXÍLIO). This research was conducted using infrastructure funded by Financiadora de Estudos e Projetos (FINEP) under the FINEP/MCTI/FNDCT/PROINFRA 2021 program (Agreement No. 0.1.22.0442.00).

## Disclosure

All authors read and approved the final manuscript. All authors are accountable for the content and conclusions of the manuscript.

## Ethics Statement

The authors have nothing to report.

## Conflicts of Interest

The authors declare no conflicts of interest.

## Supporting information


**Supporting Information** Additional supporting information can be found online in the Supporting Information section. Table S1: Daily average total health index (HI) scores of Galleria mellonella larvae injected with various fungal suspensions or with saline solutions (PBS or IPS) as controls. Table S2:. Daily average scores for each health category of Galleria mellonella larvae injected with various fungal suspensions or with saline solutions (PBS or IPS) as controls. Table S3:. Peptides lethal doses against Candida clinical fungal strains. Table S4:. Daily average scores for each category of Galleria mellonella larvae injected with saline (PBS or IPS) and subsequently treated with peptides. Table S5: Gehan–‐Breslow–‐Wilcoxon and Mantel–‐Cox tests′’ pP values results from efficacy assays according to Figure 4 data. ‐, not applied; (ns,) not significative. (‐) not applied. Table S6:. Daily average scores for each health category of Galleria mellonella larvae infected with Candida FLD100 and treated with peptides. Figure S1:. Design of bioinspired peptides derived from VuDef1. Figure S2:. Comparison of the hemocytes number in the hemolymph of Galeria mellonella injected with IPS and PBS.

## Data Availability

The data that support the findings of this study are available from the corresponding author upon reasonable request.
