# Supplementary material for: In Vivo Evaluation of Efficacy and Toxicity of Bioinspired Peptides With Antimicrobial Properties Against Fungal Infection in Galleria mellonella Larvae
Source: Int J Microbiol. 2026 Jul 11;2026:7272293. doi: 10.1155/ijm/7272293 (PMC13355496; doi:10.1155/ijm/7272293)

Supplementary material

Figure S1. Design of bioinspired peptides derived from VuDef1.
The schematic illustrates the native γ-core region of VuDef1 (in blue) and the amino acid substitutions used to generate the DD, RR, D-RR (the D-enantiomer of RR), and WR variants. Hydrophobicity was calculated using the Wimley–White scale, where more positive values indicate greater hydrophilicity (Toledo et al., 2021).


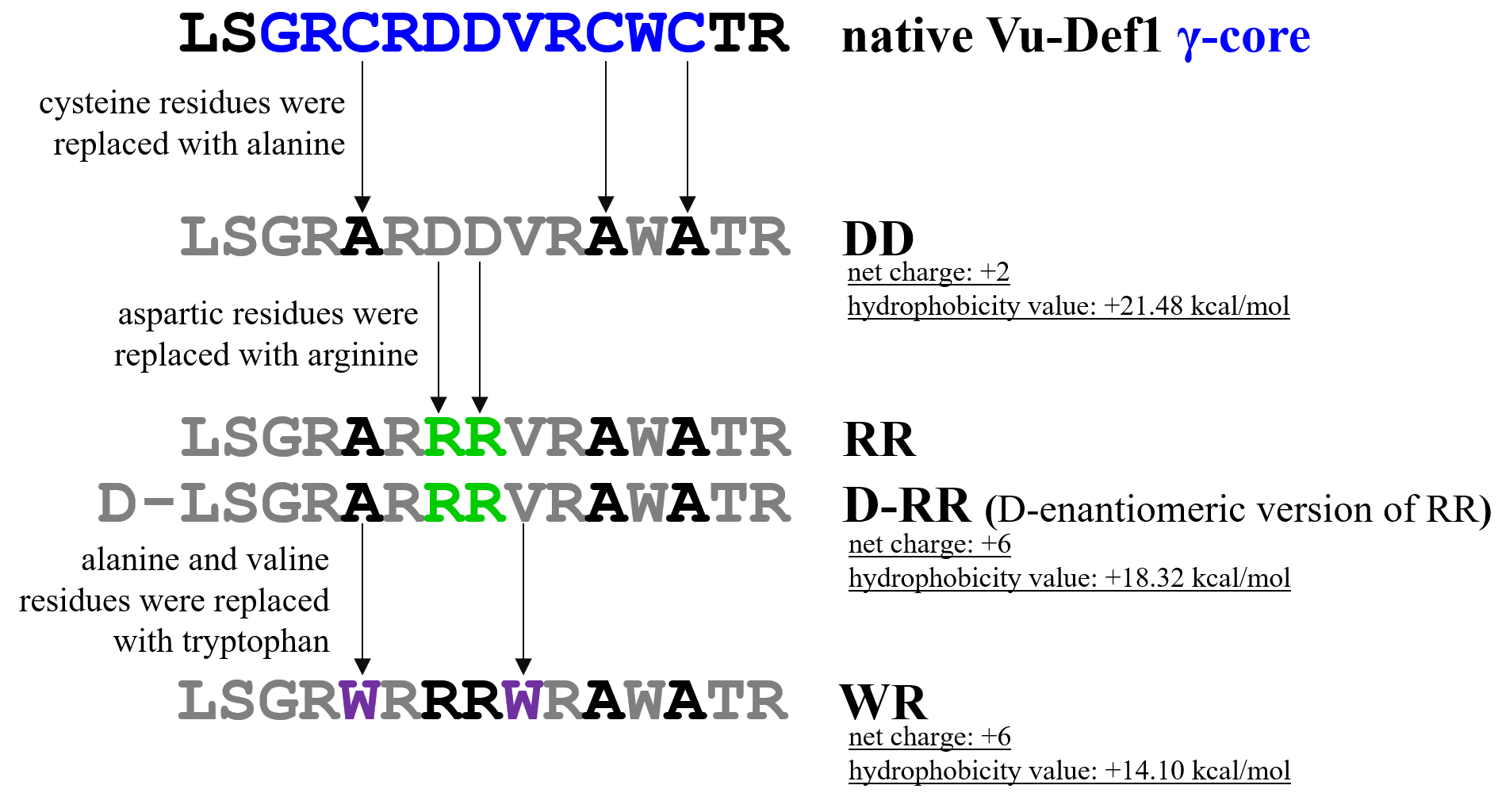


Table S1. Daily average total health index (HI) scores of *Galleria mellonella* larvae injected with various fungal suspensions or with saline solutions (PBS or IPS) as controls. The table represents the mean HI scores recorded throughout the infection period, which reflect the larvae’s health status. Scores are visually represented by color coding: green for HI > 60%, yellow for HI between 30% and 60%, red for HI < 30 %. The assay results are expressed as the average of three independent biological replicates, each containing 15 larvae per sample (n = 180 total).


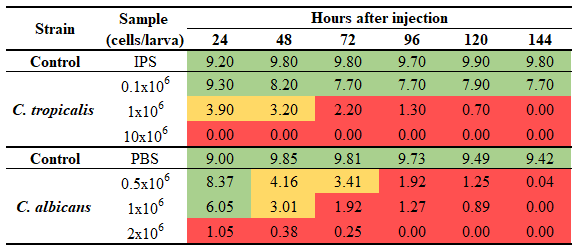


Table S2. Daily average scores for each health category of *Galleria mellonella* larvae injected with various fungal suspensions or with saline solutions (PBS or IPS) as controls. The table represents the mean scores recorded throughout the infection period, which reflect the larvae’s health status. These scores are visually represented by color coding: green for HI > 60%, yellow for HI between 30% and 60%, red for HI < 30%. The assay results are expressed as the average of three independent biological replicates, each containing 15 larvae per sample (n = 180 total).


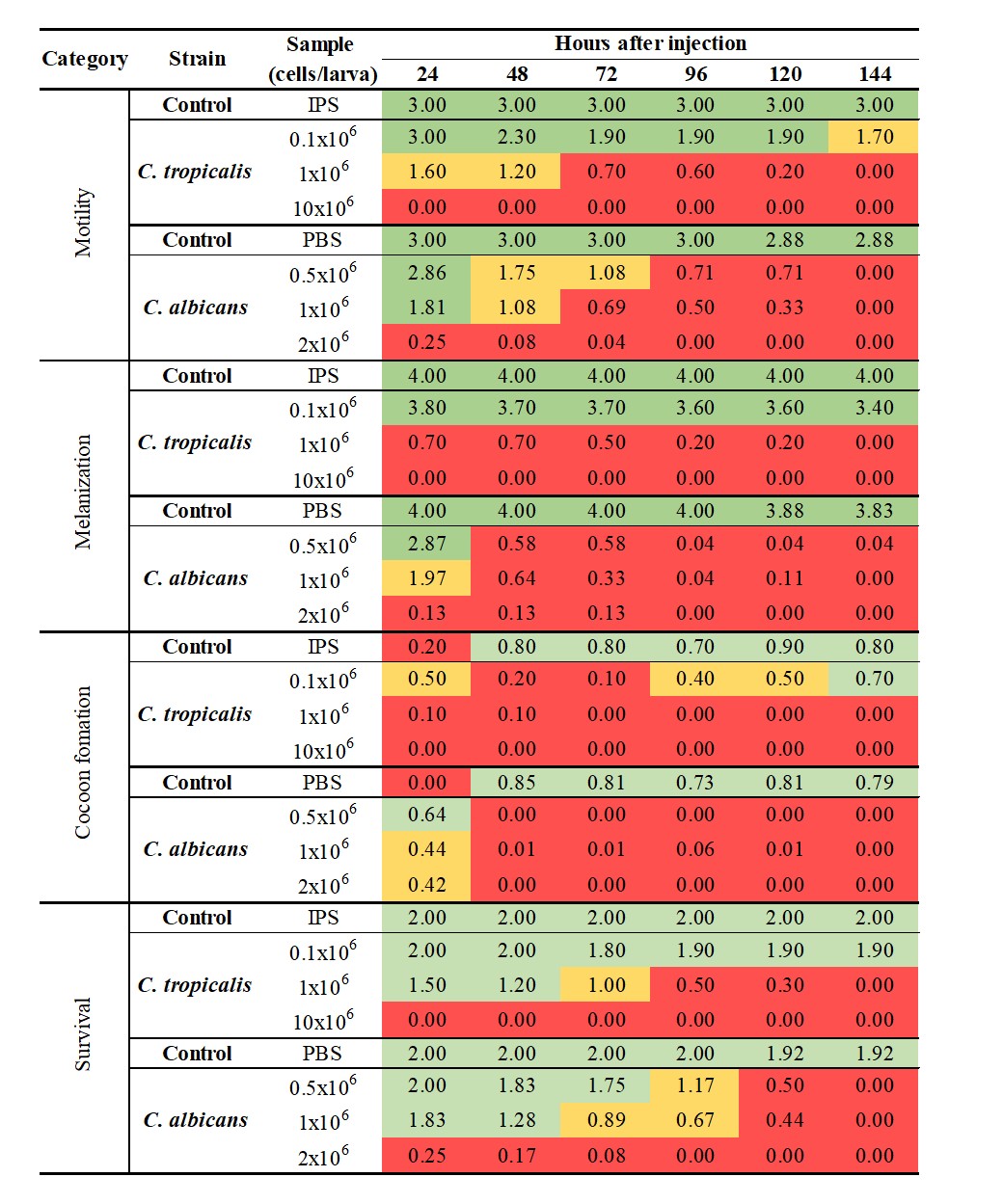


Figure S2. Comparison of the hemocytes number in the hemolymph of *Galeria mellonella* injected with IPS and PBS. Data were taken from Figure 1C (n = 5). Statistical analysis was performed using Two-Way ANOVA. Specifically, the test indicated no statistically significant difference (ns) in hemocyte counts when comparing the samples injected in IPS or PBS (Column Factor P = 0.1923, Row Factor P = 0.3994, Two-Way ANOVA).

**
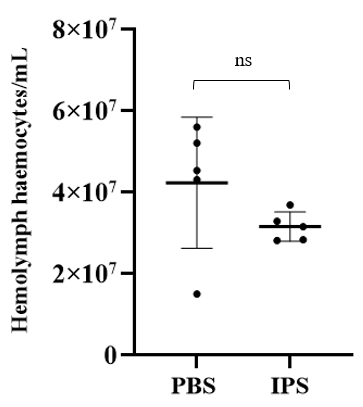
**

Table S3. Peptides lethal doses against *Candida* clinical fungal strains. The assay results are expressed as the average of three independent biological replicates, with each replicate consisting of 3 samples (n = 9 total).

**
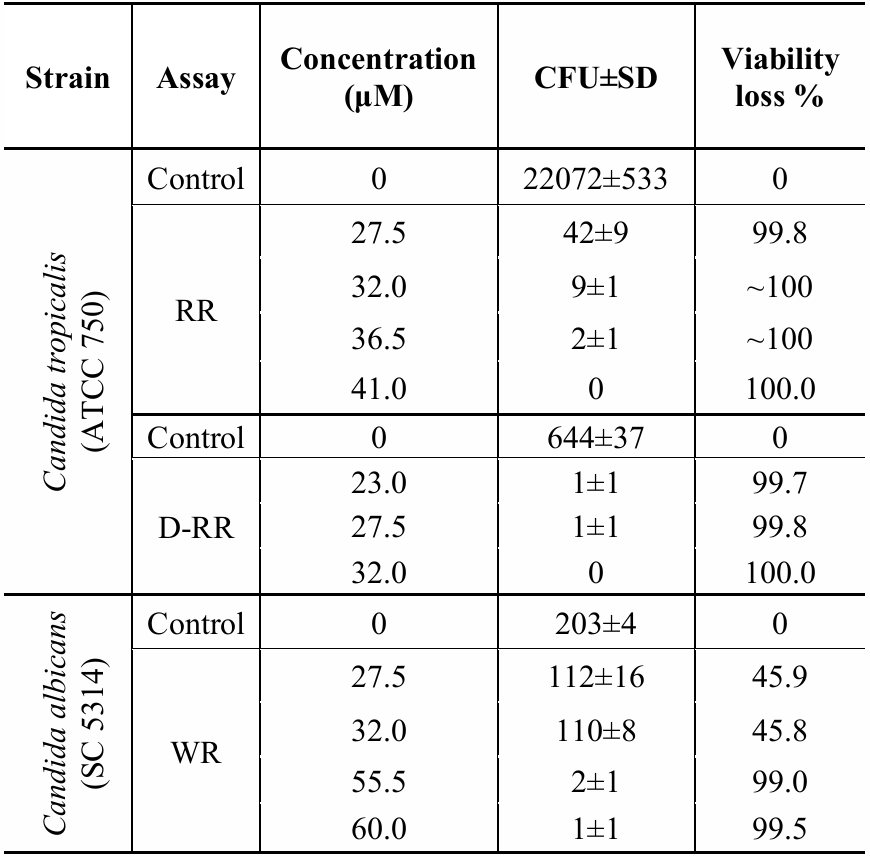
**

Table S4. Daily average scores for each category of *Galleria mellonella* larvae injected with saline (PBS or IPS) and subsequently treated with peptides. The table presents the mean health scores recorded throughout the infection period, which are proportional to larval health status. Scores are visually represented by color coding: green for HI > 60%, yellow for HI between 30% and 60%, red for HI < 30%. The assay results are expressed as the average of three independent biological replicates, with each replicate consisting of 5 larvae per sample (n = 105 total).


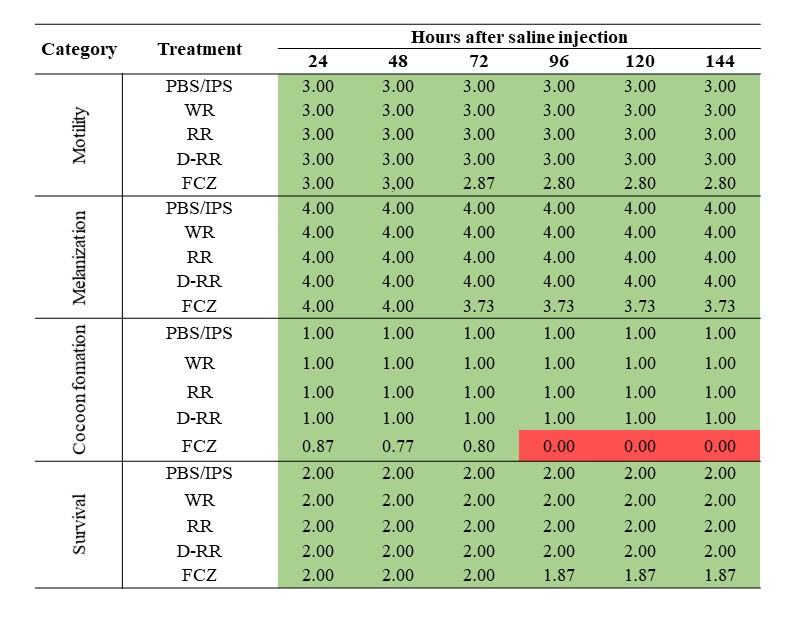


Table S5. Gehan-Breslow-Wilcoxon and Mantel-Cox tests’ P values results from efficacy assays according to Figure 4 data. (ns) not significative. (-) not applied.

| **Treatment** | ***C. tropicalis*** | ***C. tropicalis***  **+ RR** | ***C. tropicalis***  **+ D-RR** | ***C. tropicalis***  **+ FCZ** | ***C. albicans***  **+ WR** | ***C. albicans***  **+ FCZ** |
| --- | --- | --- | --- | --- | --- | --- |
| ***C. tropicalis*** | - | < 0.0001 | < 0.0001 | < 0.001 | - | - |
| ***C. tropicalis*+RR** | - | - | ns | ns | - | - |
| ***C. tropicalis*+D-RR** | - | - | - | ns | - | - |
| ***C. albicans*** | ns | - | - | - | < 0.0001 | ns |
| ***C. albicans*+WR** | - | ns | ns | - | - | 0.0001 |
| ***C. albicans*+FCZ** | - | - | - | ns | - | - |

Table S6. Daily average scores for each health category of *Galleria mellonella* larvae infected with *Candida* FLD_100_ and treated with peptides. The table presents mean scores recorded throughout the infection period, reflecting the larvae’s health status. Scores are visually represented by color coding: green for HI > 60%, yellow for HI between 30%. The assay results are expressed as the average of three independent biological replicates, with each replicate consisting of 5 larvae per sample (n = 105 total).


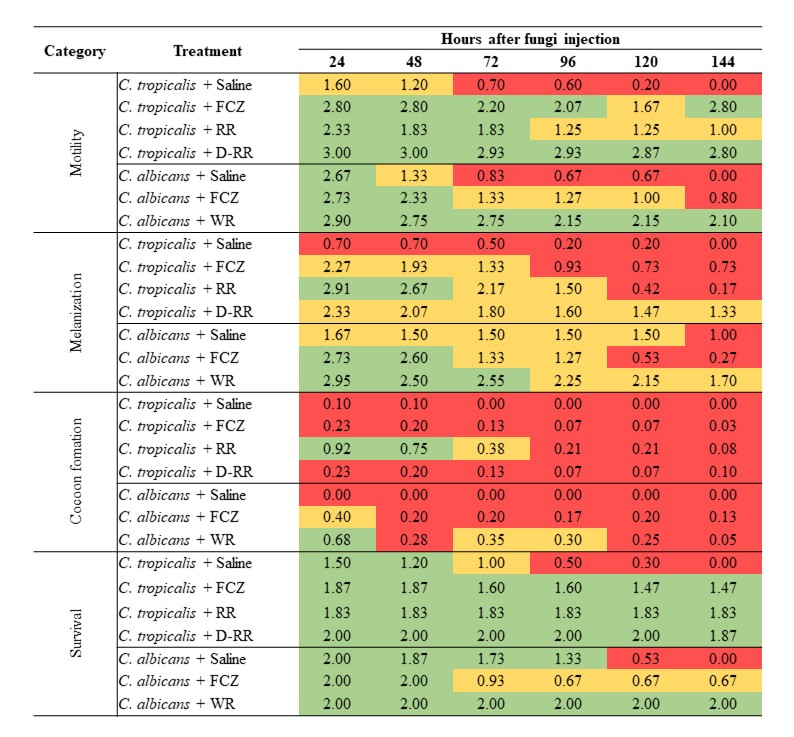

Supplement: Supplementary file 1 — Supporting Information Additional supporting information can be found online in the Supporting Information section. Table S1: Daily average total health index (HI) scores of Galleria mellonella larvae injected with various fungal suspensions or with saline solutions (PBS or IPS) as controls. Table S2:. Daily average scores for each health category of Galleria mellonella larvae injected with various fungal suspensions or with saline solutions (PBS or IPS) as controls. Table S3:. Peptides lethal doses against Candida clinical fungal strains. Table S4:. Daily average scores for each category of Galleria mellonella larvae injected with saline (PBS or IPS) and subsequently treated with peptides. Table S5: Gehan–‐Breslow–‐Wilcoxon and Mantel–‐Cox tests′’ pP values results from efficacy assays according to Figure 4 data. ‐, not applied; (ns,) not significative. (‐) not applied. Table S6:. Daily average scores for each health category of Galleria mellonella larvae infected with Candida FLD100 and treated with peptides. Figure S1:. Design of bioinspired peptides derived from VuDef1. Figure S2:. Comparison of the hemocytes number in the hemolymph of Galeria mellonella injected with IPS and PBS. [file IJM-2026-7272293-s001.doc]
